# Purification, Structural Characterization and Immunomodulatory Effects of Polysaccharides from *Amomum* *villosum* Lour. on RAW 264.7 Macrophages

**DOI:** 10.3390/molecules26092672

**Published:** 2021-05-02

**Authors:** Yang Zhou, Chunguo Qian, Depo Yang, Cailin Tang, Xinjun Xu, E-Hu Liu, Jingtang Zhong, Longping Zhu, Zhimin Zhao

**Affiliations:** 1 School of Pharmaceutical Sciences, Sun Yat-Sen University, Guangzhou 510006, China; zhouy596@mail2.sysu.edu.cn (Y.Z.); qianchg@mail2.sysu.edu.cn (C.Q.); lssydp@mail.sysu.edu.cn (D.Y.); tangclin@mail2.sysu.edu.cn (C.T.); xxj2702@sina.com (X.X.); zhlongp@mail.sysu.edu.cn (L.Z.); 2 Guangdong Technology Research Center for Advanced Chinese Medicine, Guangzhou 510006, China; 3 State Key Laboratory of Natural Medicines, School of Traditional Chinese Pharmacy, China Pharmaceutical University, Nanjing 210009, China; liuehu2011@163.com; 4 Yayisan Chinese Herbs Plantation Ltd., Heyuan 517428, China; zhaozhimin1978@hotmail.com

**Keywords:** polysaccharide, *Amomum villosum* Lour., structural analysis, immunomodulatory activity

## Abstract

*Amomum Villosum* Lour. (*A. villosum*) is a folk medicine that has been used for more than 1300 years. However, study of the polysaccharides of *A. villosum* is seriously neglected. The objectives of this study are to explore the structural characteristics of polysaccharides from *A. villosum* (AVPs) and their effects on immune cells. In this study, the acidic polysaccharides (AVPG-1 and AVPG-2) were isolated from AVPs and purified via anion exchange and gel filtration chromatography. The structural characteristics of the polysaccharides were characterized by methylation, HPSEC-MALLS-RID, HPLC, FT-IR, SEM, GC-MS and NMR techniques. AVPG-1 with a molecular weight of 514 kDa had the backbone of → 4)-α-d-Glc*p*-(1 → 3,4)-β-d-Glc*p*-(1 → 4)-α-d-Glc*p*-(1 →. AVPG-2 with a higher molecular weight (14800 kDa) comprised a backbone of → 4)-α-d-Glc*p*-(1 → 3,6)-β-d-Gal*p*-(1 → 4)-α-d-Glc*p*-(1 →. RAW 264.7 cells were used to investigate the potential effect of AVPG-1 and AVPG-2 on macrophages, and lipopolysaccharide (LPS) was used as a positive control. The results from bioassays showed that AVPG-2 exhibited stronger immunomodulatory activity than AVPG-1. AVPG-2 significantly induced nitric oxide (NO) production as well as the release of interleukin (IL)-6 and tumor necrosis factor alpha (TNF-α), and upregulated phagocytic capacities of RAW 264.7 cells. Real-time PCR analysis revealed that AVPG-2 was able to turn the polarization of macrophages to the M1 direction. These results suggested that AVPs could be explored as potential immunomodulatory agents of the functional foods or complementary medicine.

## 1. Introduction

*Amomum villosum* Lour. (Zingiberaceae, *A. villosum*) has been used as a traditional Chinese medicine for the treatment of gastrointestinal diseases for more than 1300 years [1]. *A. villosum* is widely distributed in South and East Asia [2]. Accumulating studies demonstrated that the major bioactive components of *A. villosum* included volatile oils, flavonoids, terpenoids and polysaccharides, which showed anti-inflammation, anti-oxidant, anti-ulceration and anti-microbial activities [3,4,5,6,7,8,9].

Polysaccharides are macro-biomolecules that display a wide spectrum of biological and pharmacological activities, such as anti-oxidative, immunomodulatory, anti-tumor and hypoglycemic effects [10,11,12,13,14]. However, there are only a few studies concerning *A. villosum* polysaccharides (AVPs). Studies of AVPs have focused mainly on the effect of extraction methods on properties, the optimal parameters for the extraction of AVPs by ultrasonic assisted extraction, the anti-oxidant activities in vitro and in vivo, and the immunostimulatory activities of crude polysaccharides [15,16,17]. Furthermore, almost all samples used in those studies come from their geo-authentic habitat, Yangchun, which is located in the southwest of Guangdong province [18]. However, on the market, *A. villosum* mostly comes from Yunnan province because the production from Yangchun cannot meet the increasing demand [19]. Therefore, a thorough investigation into the structure, biological activities and structure-activity relationship of purified AVPs from other regions is required.

In the present study, *A. villosum* from Yunnan province was analyzed and two fractions (named AVPG-1 and AVPG-2) were purified from it. Their structural characteristics, including molecular weight, monosaccharide composition, surface morphology, triple helical conformation and glycosyl linkages, were clarified. Furthermore, the in vitro immunomodulatory activities of the two polysaccharides were evaluated using RAW 264.7 macrophages as a model. The results of the present study could provide a better understanding of the structure-bioactivity relationship of polysaccharides and pave the road for development of new immunomodulatory agents.

## 2. Results and Discussion

### 2.1. Preparation and Physicochemical Features of Polysaccharides

**Figure 1:**
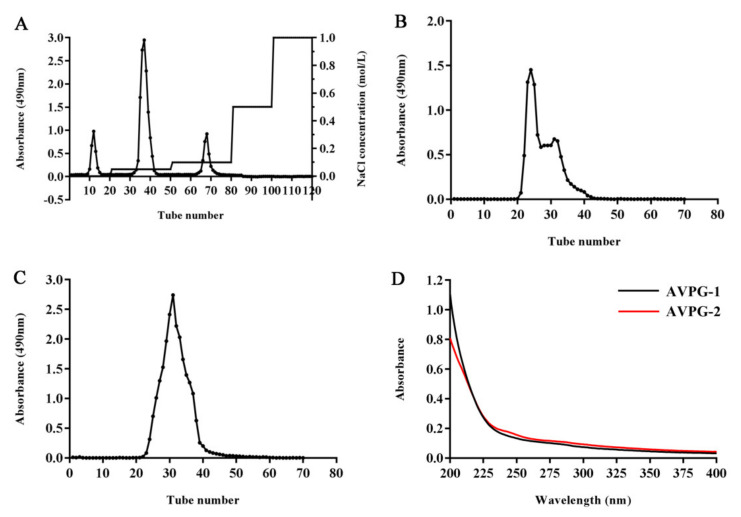
Elution curve of *A. villosum* polysaccharides (AVPs) on DEAE-52 column (**A**). Elution curve of AVPG-1 (**B**) and AVPG-2 (**C**) on Sephadex G-100 gel chromatography column. Ultraviolet (UV) spectra (**D**) of AVPG-1 and AVPG-2.

The crude polysaccharides were obtained through hot-water extraction, followed by ethanol precipitation, deproteinization and lyophilization. AVPs were purified by using DEAE-cellulose 52 (Figure 1A) and Sephadex G-100 gel filtration chromatography to obtain AVPG-1 (Figure 1B) and AVPG-2 (Figure 1C). The yields of AVPG-1 and AVPG-2 were 4.19% and 6.27%, respectively.

AVPG-1 contained a higher content of total carbohydrate (88.74% vs. 64.06%) and protein (0.18% vs. 0.14%), while AVPG-2 contained higher contents of uronic acid (28.04% vs. 7.21%) and sulfate (1.06% vs. 0.69%). It was reported that many biological activities, e.g., anti-oxidant activity, immunomodulatory activity and anti-tumor activity, were related to the molecular weight, sulfate content and uronic acid content of polysaccharides [20,21]. Moreover, the content of uronic acid was more important for anti-tumor activity than sulfate and molecular weight [22]. Both the purified polysaccharides contained a very low protein content, which was further demonstrated by ultraviolet (UV) spectrometry. The scanned UV spectra showed no absorption at 260 and 280 nm (Figure 1D) implying that neither nucleic acids nor proteins were detected in both fractions.

The endotoxin contamination existed in samples may lead to false positives in immunostimulatory assays [23]. Results of endotoxin test showed that there were low contents of endotoxin existed in AVPG-1 (0.139 EU/mL) and AVPG-2 (0.126 EU/mL). The concentrations of endotoxin in samples were lower than the endotoxin limit for a medical device (0.5 EU/mL) [24]. In addition, the macrophages were not so sensitive to react to LPS (1 ng/mL) [25]. In summary, the effect of AVPG-1 and AVPG-2 on macrophages is likely independent of any potential contaminating endotoxin.

### 2.2. Structural Characterization of Polysaccharides

#### 2.2.1. Monosaccharide Composition Identification

**Figure 2:**
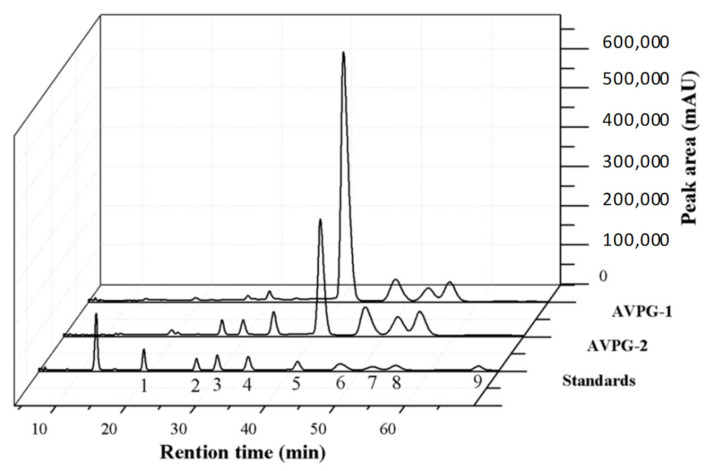
The compositions of AVPG-1, AVPG-2 and nine monosaccharide standards detected by high performance liquid chromatography (HPLC). Peak identity: (1) mannose, (2) rhamnose, (3) glucuronic acid, (4) galacturonic acid, (5) glucose, (6) galactose, (7) xylose, (8) arabinose and (9) fucose.

As shown in Figure 2, AVPG-1 is mainly comprised of glucose (73.11%), galactose (10.29%), xylose (6.21%), arabinose (8.83%) and minor glucuronic acid (1.57%). AVPG-2 is mainly comprised of rhamnose (3.11%), glucose (40.23%), galactose (17.82%), xylose (12.53%), arabinose (15.81%), glucuronic acid (3.99%) and galacturonic acid (6.51%). The monosaccharide composition of the two polysaccharides was similar. Glucose played a dominant role in all kinds of monosaccharides. The major difference between them was that AVPG-2 had a higher ratio of uronic acid than AVPG-1, which coincided with the results of the *m*-hydroxybiphenyl colorimetric test.

#### 2.2.2. Determination of Homogeneity and Molecular Weight

**Figure 3:**
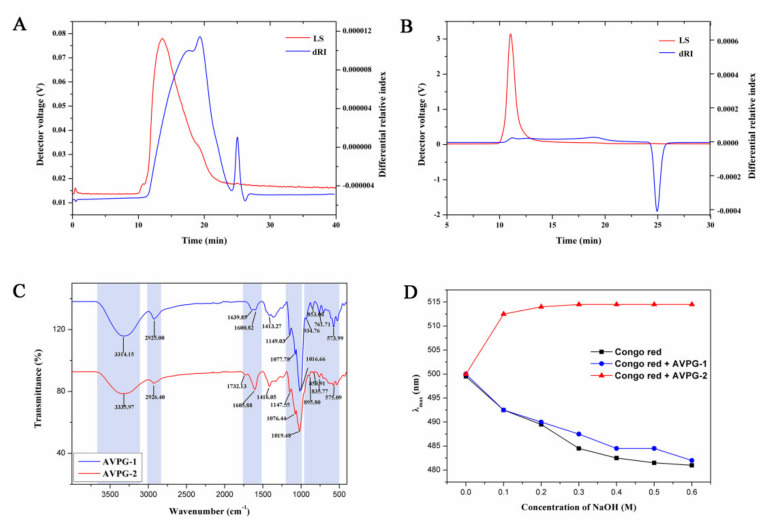
High performance size exclusion chromatography coupled with multi-angle laser light scattering and refractive index detector (HPSEC-MALLS-RID) chromatographic profile of the molar mass distribution of AVPG-1 (**A**) and AVPG-2 (**B**). Fourier transform infrared (FT-IR) spectroscopy of AVPG-1 and AVPG-2 (**C**). Maximum absorption wavelength of Congo red, Congo red + AVPG-1 or AVPG-2 at different concentrations of sodium hydroxide solution (**D**).

Biological activities of polysaccharides are closely related to their molecular weights [26]. Hence, choosing a suitable method to measure the molecular weights of polysaccharides is very important. Both AVPG-1 and AVPG-2 showed a single, sharp and symmetrical peak on the high-performance gel permeation chromatography (HPGPC) chromatogram. However, their molecular weights were too high to be out of the range of linearity. Here, high performance size exclusion chromatography coupled with multi-angle laser light scattering and refractive index detector (HPSEC-MALLS-RID) with no need of standards was used. The number-average molecular weight (*M*_n_) and weight-average molecular weight (*M*_w_) for AVPG-1 were measured as 3.48 × 10^5^ Da and 5.14 × 10^5^ Da, and for AVPG-2 were 1.15 × 10^7^ Da and 1.48 × 10^7^ Da, respectively. The value of *M*_w_/*M*_n_, which is defined as the polydispersity index (PDI), represented the uniformity of particle size distribution (PDI = 1.0 of monodispersed particles) [27]. The results indicated that the distribution of molecular weight of AVPG-1 or AVPG-2 was symmetric and unimodal because only one peak was observed (Figure 3A,B) with a relatively small PDI value of 1.47 or 1.29, respectively. As a result, either AVPG-1 or AVPG-2 was a homogeneous polysaccharide.

The *M*_w_ of polysaccharides from *A. villosum* in this study was quite different from what was reported previously [15]. The possible reasons may be the discrepancy of origin and extraction methods. There were many studies suggesting that ultrasonic-assisted extraction would decrease the molecular weight of polysaccharides [28] and hot-water extraction would make polysaccharides particles gather into large aggregates [29].

#### 2.2.3. Fourier Transform Infrared (FT-IR) Spectroscopy Analysis

Infrared spectroscopy is usually used to analyze the functional groups, monosaccharide types and glycosidic linkages of polysaccharides [30]. The FT-IR spectra of AVPG-1 and AVPG-2 are shown in Figure 3C. The FT-IR spectra could be divided into five fractions (**A**, **B**, **C**, **D** and **E**). The broad and intense peak of fraction **A** between 3600 cm^−1^ and 3200 cm^−1^ (AVPG-1: 3314.15 cm^−1^, AVPG-2: 3335.97 cm^−1^) was attributed to the stretching vibrations of hydroxyl groups (O-H) [31]. The small peaks at 2925.00 cm^−1^ and 2926.40 cm^−1^, which belonged to fraction **B**, were due to the bending vibration of C-H of the methyl groups [32]. In fraction **C**, the peaks centered at 1650–1600 cm^−1^ were caused by C=O stretching of free COO-, confirming the presence of uronic acid in AVPG-1 and AVPG-2. In addition, there was a specific peak at 1732.13 cm^−1^ shown in the spectrum of AVPG-2, which belonged to C=O stretching of methyl-esterified COO- (1760–1730 cm^−1^) [33]. Generally, the peaks of fraction **D** between 1200 cm^−1^ and 1000 cm^−1^ were called “finger-print” region of polysaccharides. In this region, multiple stretching vibrations came from asymmetric O-C-O (glycosidic linkages), C-C-O (sugar rings) and C-C-O (side groups) [34]. Three peaks were observed of AVPG-1(1149.03 cm^−1^, 1077.78 cm^−1^ and 1016.66 cm^−1^) and AVPG-2 (1147.55 cm^−1^, 1076.44 cm^−1^ and 1019.48 cm^−1^), respectively, signifying they were polysaccharides. The area of fraction **E** below 1000 cm^−1^ offers more information about the sophisticated structure [31]. The absorption peak at 853.08 cm^−1^ indicated that AVPG-1 mainly contained the α configuration [33]. The characteristic absorptions at 934.76 cm^−1^, 853.08 cm^−1^ and 761.71 cm^−1^ suggested that AVPG-1was a d-glucopyranose derivative [35]. As for AVPG-2, the weak absorption bands at 895.80 cm^−1^, 853.91 cm^−1^ and 835.77 cm^−1^ implied that α- and β- glycosidic linkages existed simultaneously [31]. In addition, peak intensities in the region of 1658–1650 cm^−1^ and 1555–1530 cm^−1^ were not detected indicating free of protein contamination [34]. These results further verified that AVPG-1 and AVPG-2 were acidic polysaccharides.

#### 2.2.4. Determination of Triple Helical Structure

Polysaccharides with a triple-helix conformation can form a specific complex with Congo red in an alkaline solution [36], and the maximum absorption wavelength (*λ*_max_) of the complex would shift to a longer wavelength. As shown in Figure 3D, an obvious red-shift of the *λ*_max_ was observed after forming the Congo red-AVPG-2 complex and then the tendency stabilized with increasing concentrations of NaOH solution (0–0.5 mol/L), while the *λ*_max_ of both Congo red and the Congo red-AVPG-1 complex decreased under the same conditions. The results suggested that AVPG-2 possessed a triple-helical conformation, while AVPG-1 did not.

#### 2.2.5. Surface Microscopic Analysis

**Figure 4:**
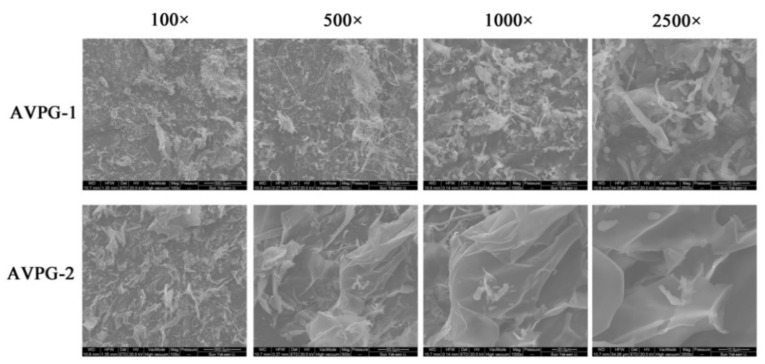
Scanning electron microscope (SEM) images of AVPG−1 and AVPG−2 (at ×100, ×500, ×1000 and ×2500 magnification).

Figure 4 shows the scanning electron microscopy (SEM) images of AVPG-1 and AVPG-2 at magnifications of 100×, 500×, 1000× and 2500×. The two polysaccharides displayed obvious variations in size and shape. AVPG-1 was gathered by a lot of particles similar to rod- and ellipsoid-shape. SEM observations revealed that the surface morphology of AVPG-2 presented a surface with a curled, tight and sheet-like appearance. In addition, few small particles were observed among the sheets. The tight and sheet-like appearance of AVPG-2 demonstrated that there might be strong interaction forces because of the highly branched conformation or electrostatic interactions between the polysaccharides [37,38].

#### 2.2.6. Methylation Analysis

Methylation analysis was performed to determine the glycosidic bonds in polysaccharides and the results were listed in Table 1. The results implied that AVPG-1 was composed of →4)-Glc*p*-(1→, →3,4)-Glc*p*-(1→, →4)-Gal*p*-(1→, Xyl*p*-(1→ and Ara*f*-(1→. AVPG-2 was composed of →4)-Glc*p*-(1→, →4)-Gal*p*-(1→, Ara*f*-(1→, →6)-Glc*p*, →3)-Gal*p*-(1→, →3,6)-Gal*p*-(1→, →5)-Ara*f*-(1→, →4)-Xyl*p*-(1→ and Rha*p*-(1→.

#### 2.2.7. Nuclear Magnetic Resonance (NMR) Analysis

**Figure 5:**
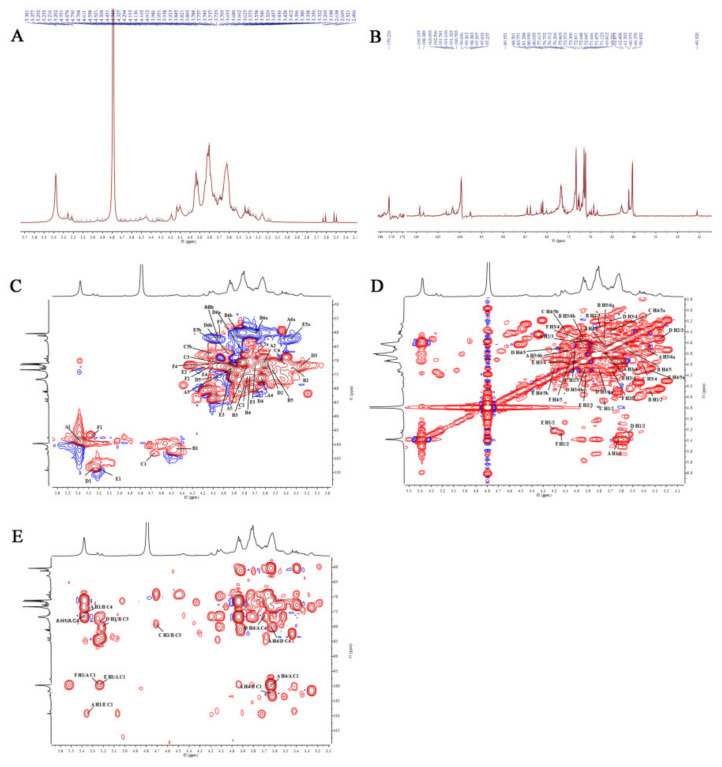
The nuclear magnetic resonance (NMR) spectra of AVPG-1 in deuterium oxide (D_2_O): (**A**) ^1^H spectrum; (**B**) ^13^C NMR spectrum; (**C**) Heteronuclear single-quantum coherence (HSQC) spectrum; (**D**) ^1^H-^1^H correlated spectroscopy (^1^H-^1^H COSY) spectrum; and (**E**) Heteronuclear multiple-bond correlation (HMBC) spectrum.

**Figure 6:**
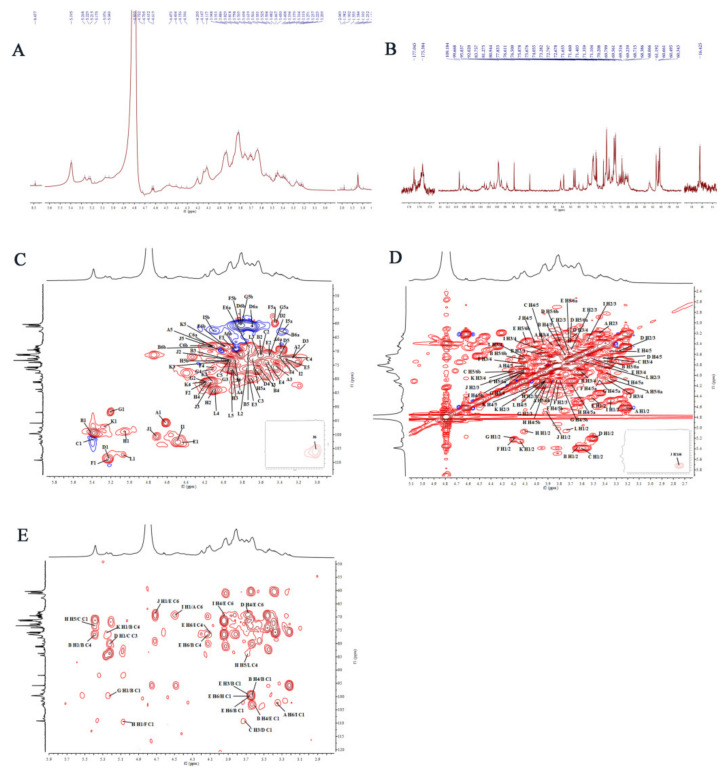
The NMR spectra of AVPG-2 in D_2_O: (**A**) ^1^H spectrum; (**B**) ^13^C NMR spectrum; (**C**) HSQC spectrum; (**D**) ^1^H-^1^H COSY spectrum; and (**E**) HMBC spectrum.

The structural characteristics of AVPG-1 and AVPG-2 were further analyzed by NMR spectra. The 1D NMR spectra and 2D NMR spectra of the two polysaccharides are shown in Figure 5 and Figure 6. Their corresponding carbon and hydrogen signals are listed in Table 2.

The ^1^H NMR spectrum of AVPG-1 showed six anomeric signals at *δ* 5.38 ppm, *δ* 4.45 ppm, *δ* 4.71 ppm, *δ* 5.22 ppm, *δ* 5.20 ppm and *δ* 5.26 ppm (denoted as **A**, **B**, **C**, **D**, **E** and **F**, respectively). In ^13^C NMR spectrum, six signals were found in the anomeric region at *δ* 99.70 ppm, *δ* 101.61 ppm, *δ* 103.10 ppm, *δ* 108.35 ppm, *δ* 109.20 ppm and *δ* 97.60 ppm. An additional signal, assigned to uronic acid, was observed in the unesterified region at *δ* 179.22 ppm.

The heteronuclear single-quantum coherence (HSQC) correlation map from anomeric region showed C1/H1 signals at *δ* 5.38/99.70 ppm (**A**), *δ* 4.45/101.61 ppm (**B**), *δ* 4.71/103.10 ppm (**C**), *δ* 5.22/108.35 ppm (**D**), *δ* 5.20/109.20 ppm (**E**) and *δ* 5.26/97.60 ppm (**F**) (Figure 5C). The α-configurations of residues **A**, **D**, **E** and **F** were confirmed from the chemical shifts of anomeric protons of greater than *δ* 4.90 ppm, whereas the β-configurations of residues **B** and **C** were confirmed by the low frequency of H-1 and high frequency of C-1 signals [39]. The chemical shifts of H-1/H-2, H-2/H-3 and H-3/H-4 were observed at *δ* 5.38/3.62 ppm, *δ* 3.62/3.95 ppm and *δ* 3.95/3.64 ppm in ^1^H–^1^H correlated spectroscopy (^1^H-^1^H COSY) spectrum, thus the chemical shifts of H-1 to H-4 in residue **A** were assigned to be *δ* 5.38 ppm, *δ* 3.62 ppm, *δ* 3.95 ppm and *δ* 3.64 ppm, respectively. The H-5 and H-6 proton chemical shifts for residue **A** were obtained based on ^1^H-^1^H COSY and HSQC spectra. According to the ^1^H NMR assignment, their corresponding carbon signals from C-1 to C-6 were identified from HSQC spectra. Combined with literature data [40] and methylation analysis, the signals derived from residue **A** were successfully assigned to →4)-α-d-Glcp-(1→. Likewise, the assignment of chemical shifts of residues **B** to **F** abided by the same routine [41,42,43,44], as shown in Table 2. The downfield shifting of carbon signal of C-4 suggested a substitution on free hydroxyl group of C-4 of residues **A**, **B** and **D**. The downfield shifting of carbon signal of C-3 suggested a substitution on free hydroxyl group of C-3 of residues **B [45]**. Therefore, residues **A** to **F** were confirmed as →4)-α-d-Glc*p*-(1→, →3,4)-β-d-Glc*p*-(1→, β-d-Xyl*p*-(1→, →4)-α-d-Gal*p*-(1→, α-l-Ara*f*-(1→ and α-d-Glc*p*A-(1→.

Subsequently, the heteronuclear multiple-bond correlation (HMBC) spectra were analyzed for the linkage sites and sequence among the sugar residues. The strong signal at *δ* 5.38/76.14 ppm (**A**H1/**A**C4) showed that C-4 of residue **A** was linked to O-1 of residue **A**. Similarly, the other cross signals were listed in Table S1. The HMBC spectrum suggested the sequences among the sugar residues of AVPG-1 are as follows: AC_4_ → AO_1_, AC_1_ → AO_4_, BC_4_ → AO_1_, BC_1_ → AO_4_, AC_4_ → DO_4_, BC_3_ → CO_1_, DC_4_ → AO_4_, EC_1_ → AO_1_, BC_3_ → DO_1_, AC_1_ → EO_1_ and AC_1_ → FO_1_. Finally, the results of monosaccharide composition, FT-IR, methylation and NMR spectra analyses suggested that AVPG-1 might be constructed by a backbone of → 4)-α-d-Glc*p*-(1 → 3,4)-β-d-Glc*p*-(1 → 4)-α-d-Glc*p*-(1 → with several side chains.

Similarly, the ^13^C NMR spectrum (Figure 6B) of AVPG-2 showed twelve anomeric carbon signals in the anomeric region, which were correlated to proton signals in the HSQC spectrum (Figure 6C). These twelve correlation peaks in the anomeric region of AVPG-2, including *δ* 4.62/95.84 ppm, *δ* 5.40/99.70 ppm, *δ* 5.39/101.63 ppm, *δ* 5.22/108.27 ppm, *δ* 4.41/103.09 ppm, *δ* 5.23/109.18 ppm, *δ* 5.20/92.01 ppm, *δ* 5.07/98.92 ppm, *δ* 4.49/102.72 ppm, *δ* 4.71/100.70 ppm, *δ* 5.26/97.61 ppm and *δ* 5.06/107.43 ppm (denoted as **A**, **B**, **C**, **D**, **E**, **F**, **G**, **H**, **I**, **J**, **K** and **L**, respectively), revealed that there were twelve sugar residues in AVPG-2. The assignment of chemical shifts of these residues were determined according to ^1^H, ^13^C, HSQC, ^1^H-^1^H COSY and HMBC spectra, as well as data reported in the literature [40,43,44,46,47,48,49] and the results of methylation analysis, as shown in Table 2. The linkage sites and sequence among the sugar residues of AVPG-2 were also analyzed according to the HMBC spectra (Figure 6E). The cross signals were listed in Table S1. The sequences among the sugar residues of AVPG-2 are as follows: BC_4_ → BO_1_, BC_1_ → BO_4_, BC_1_ → EO_3_, EC_1_ → BO_4_, IC_4_ → EO_6_, EC_6_ → IO_4_, AC_6_ → IO_1_, IC_1_ → AO_6_, EC_6_ → DO_4_, CC_3_ → DO_1_, DC_1_ → CO_3_, HC_5_ → CO_1_, FC_1_ → HO_1_, EC_6_ → JO_1_, HC_1_ → EO_6_, LC_4_ → HO_5_, BC_1_ → EO_6_, BC_4_ → KO_1_, BC_4_ → EO_6_ and BC_1_ → GO_1_. Accordingly, AVPG-2 might be a heteropolysaccharide with a backbone of → 4)-α-d-Glc*p*-(1 → 3,6)-β-d-Gal*p*-(1 → 4)-α-d-Glc*p*-(1 → with several side chains.

### 2.3. Immunomodulatory Activities

Macrophages belong to the congenital immune system and play a vital role in the innate and adaptive immune regulation. Macrophages have multiple functions such as phagocytosis, antigen presentation and production of different type of cytokines [50]. In the present study, the immunomodulatory activities of polysaccharides were assayed using RAW 264.7 cells by measuring their viability, phagocytosis, induced nitric oxide (NO) and cytokines production.

#### 2.3.1. Effects of Polysaccharides on Cell Viability and NO Production of Macrophages

**Figure 7:**
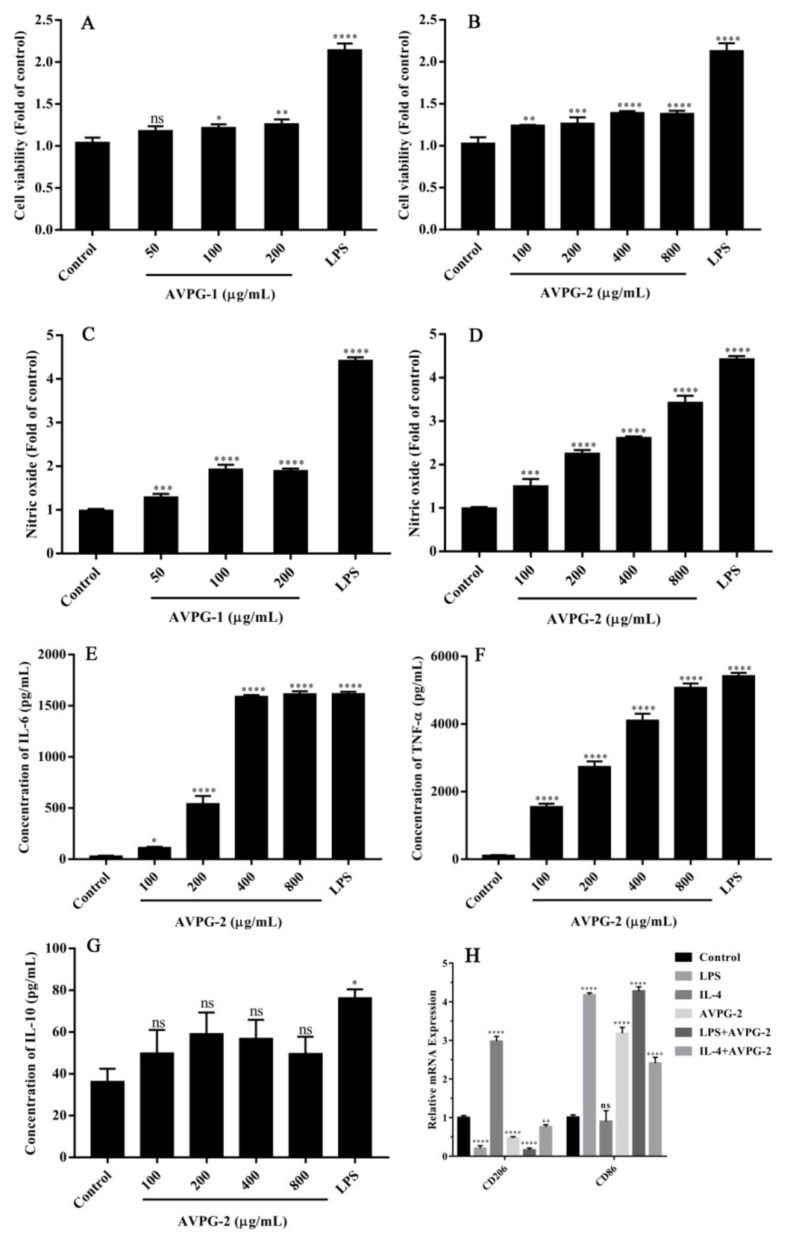
Effects of AVPG-1 and AVPG-2 on cell proliferation (**A**,**B**) and nitric oxide (NO) release (**C**,**D**) of RAW 264.7. Effects of AVPG-2 on interleukin (IL)-6 (**E**), tumor necrosis factor alpha (TNF-α) (**F**) and IL-10 (**G**) production of RAW 264.7. Effects of AVPG-2 on mRNA expression of CD86 and CD206 (**H**), as determined by real time PCR (RT-PCR). Ns represents no significant difference versus control group, * *p* < 0.05 versus control group, ** *p* < 0.01 versus control group, *** *p* < 0.001 versus control group, **** *p* < 0.0001 versus control group.

The Cell Counting Kit-8 (CCK-8) assay was used to measure the influence of AVPG-1 and AVPG-2 on the viability of RAW 264.7 cells. As shown in Figure 7A,B, both polysaccharides increased cell proliferation at the tested concentrations in comparison with the blank control. AVPG-2 exhibited a better activity of stimulating cell proliferation than that of AVPG-1.

NO is a kind of important molecules with lots of physiological functions like resisting pathogen invasion and regulating apoptosis [51]. Besides, the increased NO secretion was also commonly function as an indicator of macrophage activation, especially in the assessment of immunostimulatory activity of polysaccharides. As shown in Figure 7C,D, AVPG-1 significantly increased the NO production at concentrations of 50, 100 and 200 μg/mL (*p* < 0.05), and the amount of NO released reached the maximum when the concentration was 100 μg/mL. AVPG-2 exhibited strong stimulatory effect on RAW 264.7 cells at all concentrations. The amount of NO released by RAW 264.7 cells was close to that of the lipopolysaccharide (LPS) group when the concentration of AVPG-2 reached 800 μg/mL. Moreover, AVPG-2 induced significantly more NO production than AVPG-1. This difference might be related to the molecular weight. It was reported that molecular weight of polysaccharides was a key factor to affect NO production, and the polysaccharides with higher molecular weight induced more NO production [52].

The immunomodulatory activity of AVPG-2 was better than that of AVPG-1, as shown by the results of the cell viability and NO production assays. As a result, AVPG-2 was selected for further investigation.

#### 2.3.2. Effect of AVPG-2 on Cytokines Secretion of Macrophages

Interleukin (IL-6) is a key signaling molecule in regulating acute-phase response, that promotes the population expansion and activation of T cells and the differentiation of B cells [53]. TNF-α is one of the most important cytokines that regulates acute local or systemic immune and inflammatory responses [54]. Briefly, IL-6 and tumor necrosis factor (TNF)-α are pro-inflammatory cytokines playing important roles in the regulation of the immune response. IL-10 is secreted by a wide range of cell types and has been intensively studied due to its pleiotropic and at times seemingly contradictory effects. In this work, the secretion of IL-6, TNF-α and IL-10 were investigated by Enzyme-linked Immunosorbent Assay (ELISA) kits. Results showed that AVPG-2 significantly promoted the secretions of IL-6 and TNF-α in RAW 264.7 cells compared to the control group (*p* < 0.05) (Figure 7E,F). The effect of AVPGs on the secretion of IL-6 was comparable to that of LPS when the concentrations of AVPG-2 reached to 400 and 800 μg/mL. However, the production of IL-10 was slightly increased (Figure 7G). According to these data, we speculated that RAW 264.7 cells were activated into M1 macrophages after exposure to AVPG-2.

#### 2.3.3. Effects of AVPG-2 on Polarization of Macrophages

Macrophages can be polarized into M1 or M2 macrophages by environmental factors. M1 macrophages participate in the removal of pathogens; they secrete higher levels of pro-inflammatory cytokines such as TNF-α, IL-1α, IL-1β, IL-6, IL-12, IL-23 and cyclooxygenase (COX)-2 and low levels of IL-10. In contrast, M2 macrophages produce anti-inflammatory cytokines such as higher levels of IL-10 and transforming growth factor (TGF)-β, but little IL-12 [55]. The two sub-populations of macrophages have different functions and participate in the pathogenic process of various diseases.

To further investigate which subtypes are activated in response to AVPG-2 treatment, the transcription of CD86 and CD206, two commonly used markers of M1 macrophages and M2 macrophages were investigated [56]. LPS and IL-4 are the M1 and M2 polarization agents, respectively [57,58]. As shown in Figure 7H, LPS and IL-4 upregulated the mRNA expression of CD86 and CD206, respectively. This result indicated that the experimental models were established successfully. When RAW 264.7 cells were exposed to AVPG-2, the mRNA expression of CD86 was increased, while CD206 decreased, which was similar to LPS. When the cells were co-stimulated by IL-4 and AVPG-2, the mRNA expression of CD86 was increased, while CD206 decreased slightly, which indicated that IL-4 induced M2 polarization was reversed. This phenomenon did not occur in the co-stimulation group of LPS and AVPG-2. This experiment indicated that AVPG-2 induced M1 polarization in macrophages and it could screw the polarization of macrophages to M1 direction. The result confirmed the conjecture mentioned above.

#### 2.3.4. Effects of AVPG-2 on Phagocytic Capacity of Macrophages

**Figure 8:**
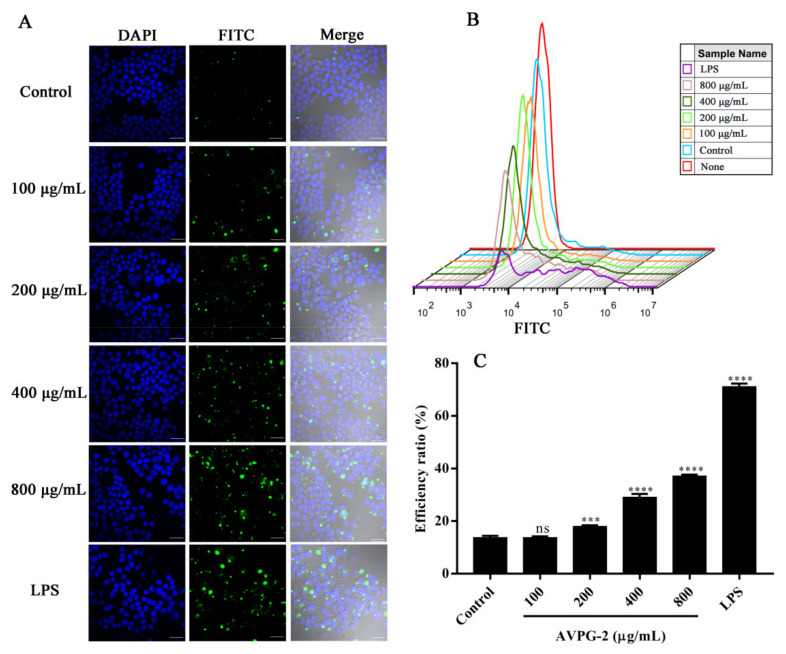
Effects of AVPG-2 on the phagocytic capacity of RAW 264.7 cells. (**A**) Images were captured by laser scanning confocal microscope (×60 magnification). Scale bars, 30 μm. The nuclei were visualized by DAPI (blue), and fluorescein isothiocyanate (FITC)-labeled *E. coli* appeared green fluorescence. (**B**) Fluorescence intensity detected by flow cytometry. (**C**) Efficiency ratio defined as percent of positive fluorescence cells in total cells. Ns represents no significant difference versus control group, *** *p* < 0.001 versus control group, **** *p* < 0.0001 versus control group.

The process for large particles engulfed by membrane protrusions is called phagocytosis [59]. Phagocytosis is a vital basis for macrophages to exert its functions in eliminating pathogens and damaged cells, and it is one of the indicators for macrophage activation. Here, we determined the effect of AVPG-2 on phagocytic capacity of RAW 264.7 cells qualitatively by using fluorescein isothiocyanate (FITC)-labeled *Escherichia coli* (*E. coli*) on a laser scanning confocal microscope. As illustrated in Figure 8A, the FITC-labeled *E. coli* (green fluorescence) were apparently engulfed into cells and outside of nucleus (blue fluorescence). A dose-dependent increase of the intensity of green fluorescence was observed in AVPG-2 treated groups. To perform a quantitative analysis of the fluorescence intensity, a flow cytometer was used. The results showed that the fluorescence intensity gradually increased with increasing concentrations of AVPG-2 (Figure 8B). The efficiency ratio, which was defined as the percentage of cells that have engulfed FITC-labeled *E. coli* [60], of positive fluorescence cells in drug groups at concentrations of 200, 400 and 800 μg/mL was significantly higher than that of control group (*p* < 0.001) (Figure 8C).

The immunomodulatory activity study indicated that AVPG-1 and AVPG-2 exhibited immunostimulatory activity and the effect of AVPG-2 was stronger than that of AVPG-1. The bioactivities of polysaccharides were closely related to its chemical structure. Growing evidence demonstrated that these structural features mainly include monosaccharide composition, molecular weight, glycosidic linkage types, the diastereomeric forms and conformation [61,62,63]. Structural analyses indicated that AVPG-2 possessed a higher molecular weight (1.48 × 10^7^ g/mol), higher uronic acid content (28.04 ± 0.46) and more glycosidic linkage types than AVPG-1. Moreover, AVPG-2 existed as a triple helix structure but AVPG-1 did not. According to previous studies, all those features (higher molecular weight [64], higher uronic acid content [65], specific glycosidic linkage types [54] and triple helix structure [26]) made a stronger immunomodulatory activity of polysaccharides. Further experiments suggested that AVPG-2 could enhance the secretion of NO, IL-6 and TNF-α by RAW 264.7 cells and slightly elevate cell viability, as well as improve phagocyte activity of macrophages. Aside from function as markers of M1 macrophages, the pro-inflammatory factors are the mediators to exert their immunomodulatory function of macrophages. For example, IL-6 plays an important role in boosting T cells trafficking to lymph nodes and to tumor sites, where they could become activated and execute their cytotoxic effector functions, respectively [66]. TNF-α could also enhance anti-tumor immunity [67]. RT-PCR analysis showed that macrophages could be polarized into M1 but not M2 phenotype upon AVPG-2 stimulation. The different macrophage populations, M1 and M2, play different roles in various processes [50]. For example, M1 macrophages are able to kill tumor cells, while M2 macrophages promote angiogenesis and tumor growth [68]. In patients with various cancers, the presence of M2 macrophages are closely correlated to poor prognosis [69]. Our results demonstrated that AVPG-2 was able to turn the polarization of macrophages to the M1 direction, which may provide additional help to slow down the progression of malignant diseases [70]. However, M1 macrophages would damage normal cells and tissues when overly induced. Keeping the balance of M1/M2 macrophage polarization is very important, because it governs the fate of an organ in inflammation or injury. AVPG-2 activated macrophages moderately in the present study. Although the bioavailability and bacteria digestion were commonly considered as limiting factor for the clinical usage of polysaccharide, accumulating studies demonstrated certain polysaccharides were able to penetrate into the gastrointestinal mucous barrier and ingest into the blood circulation. For example, a polysaccharide from *Angelica sinensis* was able to penetrate through the intestinal monolayer via endocytosis and absorb into the blood [71]. Additionally, some peptides were able to inhibit the digestive enzyme, which may provide an alternative way to promote its absorption and bioavailability [72]. Considering the plant derived polysaccharides share similar physicochemical properties, the AVPG-2 identified in our study may absorb as their original form in vivo, at least partially, and exert its immunoregulatory effects. It is possible to develop AVPG-2 into immunomodulatory agents. Further investigations should focus on the pathways related to absorption and metabolism. The signaling pathways for immune regulation also need to be clarified.

## 3. Materials and Methods

### 3.1. Materials and Reagents

The seeds of *A. villosum* were purchased from Wenshan Autonomous Prefecture, Yunnan Province, China: 2019 harvest. DEAE-52 Cellulose and Sephadex G-100 were purchased from Solarbio (Beijing, China). Monosaccharide standards and LPS were purchased from Sigma-Aldrich Chemical Co. (St., Louis, MO, USA). Deuterium oxide and trifluoroacetate acid (TFA) were purchased from Sigma-Aldrich Co. (Beijing, China). Dulbecco’s modified eagle’s medium (DMEM), fetal bovine serum (FBS), penicillin and streptomycin were purchased from Gibco Life Technologies (Grand Island, NY, USA). Chromatographic grade acetonitrile was purchased from Oceanpak (Goteborg, Sweden). The CCK-8 was acquired from Beijing Solarbio Science & Technology Co., Ltd. (Beijing, China). The Griess assay kit was obtained from Beyotime Biotechnology (Shanghai, China). Mouse ELISA kits to detect TNF-α, IL-6 and IL-10 were purchased from Neobioscience Technology Co., Ltd. (Shenzhen, China). All other reagents were of analytical grade.

### 3.2. Extraction and Purification of Polysaccharides

The seeds of *A. villosum* were pulverized into fine powder and then passed through a 40-mesh sieve. The powder (200 g) was pretreated three times with 95% ethanol at 80 °C for 3 h to remove lipids and pigments. The residue was air-dried and extracted three times with water (4 L) at 95 °C for 3 h. Subsequently, the supernatant was gathered, centrifuged at 5000 rpm for 10 min, concentrated using a vacuum rotary evaporator (Gongyi Yuhua Experimental Instrument Co., Ltd., Gongyi, China), and then mixed with four volumes of absolute ethanol overnight at 4 °C. The precipitate was collected by centrifugation at 5000 rpm for 10 min. After removing proteins by Sevag regent, the crude polysaccharides were obtained.

AVPs were applied to a DEAE Cellulose-52 column (2.6 × 30 cm) and then stepwise eluted using distilled water, followed by 0.05, 0.1, 0.5 and 1.0 M NaCl solutions at a flow rate of 1.0 mL/min. Three fractions that were eluted using distilled water, 0.05 M and 0.1 M NaCl solutions were obtained and named as AVPD-0, AVPD-1 and AVPD-2, respectively. AVPD-1 and AVPD-2 were further dialyzed using a dialysis bag (molecular weight cut-off: 3500 Da) against running water for 24 h, and then freeze-dried. Sephadex G-100 chromatography column (2.6 × 60 cm) was used to further purify the two fractions, resulting in the target polysaccharides designated as AVPG-1 and AVPG-2.

### 3.3. Preliminary Chemical Analysis

The content of carbohydrate in each sample was determined by phenol-sulfuric acid colorimetric method using glucose as the standard [73]. The content of protein was measured by the Bradford method using bovine serum albumin as the standard [74]. The content of uronic acid was analyzed by the *m*-hydroxybiphenyl colorimetric method using D-glucuronic acid as the standard [75]. The content of sulfate was tested by the barium chloride-gelatin method [76]. The content of endotoxin in the samples was determined by the end-point chromogenic tachypleus amebocyte lysate (TAL) assay kit [77]. The procedures were performed according to the manufacturer’s instructions. The samples were diluted to 800 μg/mL.

### 3.4. Determination of Homogeneity and Molecular Weight

The *M*_n_, *M*_w_ and PDI for the two polysaccharide fractions in 0.9% NaCl aqueous solution were measured using HPSEC-MALLS-RID. HPSEC-MALLS-RID measurements were carried out on a multi-angle laser light scattering detector (DAWN HELEOS-II, Wyatt Technology Co., Santa Barbara, CA, USA) with a separation module (Waters e2695) equipped with a TSK-Gel G4000SWXL column (300 mm × 7.8 mm, i.d., Tosoh Bioscience, Tokyo, Japan) at 35 °C. A differential refractive index detector (RID, Optilab T-rEX, Wyatt Technology Co., Santa Barbara, CA, USA) was connected simultaneously. The mobile phase was 0.9% NaCl aqueous solution and was passed through the HPSEC column at a flow rate of 0.5 mL/min. Astra software (Version 7.1.3) was utilized for data acquisition and analysis. The molecular weight was calculated using the Zimm model.

### 3.5. Monosaccharide Composition Identification

The monosaccharide composition of the polysaccharides was evaluated using HPLC according to a previously reported method with some modifications [78]. Briefly, AVPG-1 and AVPG-2 were hydrolyzed with 2 mL of 3 M TFA at 120 °C for 6 h. After removal of the TFA, the hydrolysates were dissolved in 0.8 mL deionized water, and then 0.1 mL 0.5 M 1-phenyl-3-methyl-5-pyrazolone (PMP) and 0.1 mL 0.3 M NaOH were added. The mixtures were reacted at 70 °C for 30 min. After being neutralized by adding 0.3 M HCl, the resulting solutions were extracted with chloroform three times. Finally, the sample solutions were filtered through a 0.22 μm syringe filter and analyzed by HPLC (Shimadzu Corporation, Kyoto, Japan) using a Zorbax Eclipse XDB-C18 column (4.6 × 250 mm, 5 μm, Agilent Technologies, Santa Clara, CA, USA). The optimal mobile phase consisted of 0.05 M phosphate buffer (pH 6.7, solvent A) and acetonitrile (solvent B) at a flow rate of 1 mL/min. The gradient elution was as follows: started from 16.0% B and maintained for 40 min, followed by a liner decrease to 14% B in 1 min and maintained for 13 min, and finally returned to 16% B in 1 min and then isocratic for 15 min. The column temperature was kept at 35 °C and the injection volume was 20 μL, with a detection wavelength of 250 nm. Glucose (Glc), mannose (Man), galactose (Gal), rhamnose (Rha), xylose (Xyl), arabinose (Ara), fucose (Fuc), glucuronic acid (GlcA) and galacturonic acid (GalA) were used as monosaccharides standards.

### 3.6. UV Spectroscopy and FT-IR Spectroscopy Analysis

The samples were dissolved in deionized water at a concentration of 1.0 mg/mL, and the UV spectra of AVPG-1 and AVPG-2 were recorded in the wavelength range of 200–400 nm using a UV-vis spectrophotometer (UV-2600, Shimadzu Corporation, Kyoto, Japan).

The FT-IR spectra were obtained using a Fourier transform infrared spectrophotometer (UATR Two, PerkinElmer, The Netherlands) in the range of 4000–400 cm^−1^.

### 3.7. Determination of Triple-Helical Structure

The Congo red test was used to determine the conformation of AVPG-1 and AVPG-2 according to a previous report with some modifications [79]. Briefly, each sample solution (1.0 mL, 1.0 mg/mL) was thoroughly mixed with 1.0 mL of Congo red solution (80 μM). NaOH solution (1.0 M) was gradually added into the mixture until the final concentration of NaOH reached to 0, 0.1, 0.2, 0.3, 0.4, 0.5 or 0.6 M. After standing for 10 min at room temperature (RT), the *λ*_max_ of the mixture was recorded by using Shimadzu UV-2600 spectrophotometer in the range of 400–600 nm.

### 3.8. Surface Microscopic Analysis

A field-emission scanning electron microscope (FESEM, Gemini500, Zeiss/Bruker, Oberkochen, Germany) was used to obtain the surface micrographs of AVPG-1 and AVPG-2. The dried samples were affixed on a specimen holder with the help of double-sided adhesive tape and coated with gold powder to make them conductive. The images were obtained at an accelerating voltage of 20.0 kV.

### 3.9. Methylation Analysis

The polysaccharides were methylated in accordance with previous method [80] to analyze the glycosidic linkage pattern. Briefly, each polysaccharide (8 mg) was dissolved in 5 mL of anhydrous dimethylsulfoxide (DMSO) and sonicated for 1 h. Next, 600 mg of powdered NaOH was added and sonicated for 1 h. Subsequently, methyl iodide (500 μL) was added in the dark and sonicated for 10 min. The process was repeated three times, and the final sonication continued for 1 h. Finally, distilled water (2 mL) was added to terminate this reaction and chloroform was used to extract the methylated polysaccharide. Complete methylation of polysaccharides was confirmed by the disappearance of –OH band (3000–3500 cm^−1^) in FT-IR spectrum (Figures S1 and S2).

The partially methylated polysaccharide was hydrolyzed with 3 M TFA at 120 °C for 6 h, reduced with NaBD_4_, and then acetylated with acetic anhydride to yield partially methylated alditol acetates (PMAA). Then, the PMAA were identified by gas chromatography-mass spectrometry (GC-MS) using an HP-5 MS fused silica capillary column. The initial column temperature was 80 °C (held for 1 min), after which the temperature was increased to 280 °C at 5 °C/min and held for 1 min. The compound that corresponded to each peak was identified by comparing to the MS data in the CCRC database (https://www.ccrc.uga.edu/specdb/ms/pmaa/pframe.html, accessed on 27 April 2021).

### 3.10. NMR Spectroscopy Analysis

AVPG-1 and AVPG-2 was dissolved in deuterium oxide (D_2_O) at 60 mg/mL, respectively. The samples were then subjected to a Bruker Avance-600 spectrometer (Bruker, Fällanden, Switzerland) to record NMR spectra at 30 °C including ^1^H NMR, ^13^C NMR, ^1^H–^1^H COSY, HSQC and HMBC. MestReNova Software was used to process the NMR data, and chemical shifts (*δ*) were expressed in ppm.

### 3.11. Immunomodulatory Activity

#### 3.11.1. Cell Culture

The RAW 264.7 murine macrophage cell line was purchased from the Cell Bank of Shanghai Institute of Biochemistry and Cell Biology (Chinese Academy of Sciences, Shanghai, China). RAW 264.7 cells were cultured in DMEM high-glucose medium supplemented with 10% FBS, 100 U/mL penicillin and 100 μg/mL streptomycin at 37 °C in a humidified atmosphere of 5% CO_2_.

#### 3.11.2. Assay of Cell Viability

The effect of AVPG-1 and AVPG-2 on cell viability was evaluated by the CCK-8 assay according to the manufacturer’s instructions. Briefly, RAW 264.7 cells (5.0 × 10^3^ cells/well) were cultured in 96-well plates for 24 h, and then treated with polysaccharides at final concentrations of 100, 200, 400 and 800 μg/mL for 24 h. An equal volume of culture medium was used as a blank control and LPS (1 μg/mL) was used as a positive control. After adding 10 μL of CCK-8 to each well, the plates were further incubated for 3 h at 37 °C. Subsequently, the optical density (OD) was read on a microplate reader (Epoch, BioTeck Instruments Inc., Winooski, VT, USA) at 450 nm. Cell viability was expressed as the ratio of absorbance values between treatments and the blank control group. The experiment was performed in triplicate.

#### 3.11.3. Assay of NO

RAW 264.7 cells (5.0 × 10^4^ cells/well) were seeded in 96-well plates for 24 h, and then stimulated by polysaccharides (100, 200, 400 and 800 μg/mL) or 1.0 μg/mL LPS (positive control). Culture medium was used as a blank control. NO secreted by macrophage RAW 264.7 cells was determined by Griess reaction according to the manufacturer’s instructions. The results were calculated as ratio of absorbance values between treatments and the blank control group.

#### 3.11.4. Determination of Cytokines

The concentrations of TNF-α, IL-6 and IL-10 released from RAW 264.7 cells were determined using ELISA kits according to the manufacturer’s instructions. The experiment was performed in triplicate.

#### 3.11.5. Real Time (RT)-PCR

RAW 264.7 cells were cultured in 6-well plates at a density of 1.1 × 10^6^ cells/well for 24 h, and then treated with LPS (1.0 μg/mL), IL-4 (20 ng/mL), polysaccharides (400 μg/mL); polysaccharides (400 μg/mL) and LPS (1.0 μg/mL); polysaccharides (400 μg/mL) and IL-4 (20 ng/mL); or culture medium (blank control) for 24 h, respectively. Total RNA was extracted using the Trizol reagent (Invitrogen, Carlsbad, CA, USA). The concentration of RNA in samples was measured by a Nanodrop 2000 ultramicro spectrophotometer (Thermo Fisher Scientific, Waltham, MA, USA). Then, the mRNA samples were reversely transcribed into complementary DNA using the HiScript II Q RT SuperMix for qPCR (Vazyme, Nanjing, China). Quantitative RT-PCR assays were conducted using the Hieff^TM^ qPCR SYBR^®^ Green Master Mix (Yeasen, Shanghai, China) and detected by the LightCycler 96 RT-PCR System (Roche, Basel, Switzerland). Glyceraldehyde 3-phosphate dehydrogenase (GAPDH) was used as an internal control. All experiments were performed in triplicate. The following primers were used: CD206 forward 5′-GTCAGAACAGACTGCGTGGA-3′, CD206 reverse 5′-AGGGATCGCCTGTTTTCCAG-3′; CD86 forward 5′-ATGGACCCCAGATGCACCA-3′, CD86 reverse CCTTTGTAAATGGGCACGGC; and GAPDH forward 5′-GGTGAAGGTCGGTGTGAACG-3′, GAPDH reverse 5′-CTCGCTCCTGGAAGATGGTG-3′.

#### 3.11.6. Assay of Macrophages Phagocytosis

Phagocytosis of target particles by RAW 264.7 cells was analyzed qualitatively using microscopy and quantitatively using flow cytometry.

RAW 264.7 cells were plated in glass bottom cell culture dishes at a density of 1 × 10^5^ cells/well and exposed to complete medium alone (blank control) or different concentrations of polysaccharides or LPS (positive control) for 24 h. After treatment, FITC-labeled *E. coli* (K-12 strain) BioParticles (Molecular Probes: E2861) were incubated with macrophages for 30 min. The final number ratio of cells to bacteria was 1:3000. The fluorescent signal of extracellular *E. coli* was removed by cold PBS, and the cells were then fixed in 4% paraformaldehyde for 20 min. Nuclei were stained by DAPI for 15 min after removing the paraformaldehyde. The excess dye solution was removed by cold PBS. Finally, macrophages phagocytosis of FITC-labeled *E. coli* was measured using laser scanning confocal microscopy (LSCM, FV 3000, Olympus, Tokyo, Japan).

RAW 264.7 cells were seeded in a 12-well plate at a density of 1 × 10^5^ cells/well. The cells were treated with polysaccharides, LPS or culture medium and FITC-labeled *E. coli* as described above. After the extracellular signals were washed out, cells were gently de-adhered by pipetting up and down, and then transferred into micro-centrifuge tubes for flow cytometry. All data were acquired on a Beckman-Coulter CytoFLEX S instrument and analyzed with FlowJo V10 software.

### 3.12. Statistical Analysis

The results were expressed as mean ± standard deviation (SD) of three replicates. One-way ANOVA with Dunnett post hoc test was used for the statistical analysis. Significant difference was considered when * *p* < 0.05, ** *p* < 0.005, *** *p* < 0.001, **** *p* < 0.0001, and all the analyses were performed using GraphPad Prism^®^ version 7.0 (GraphPad Software, San Diego, CA, USA).

## 4. Conclusions

In summary, two polysaccharides of *Amomum villosum* Lour. were successfully isolated and purified, and their structural characterization and immunomodulatory activities were explored. The average molecular weight of AVPG-1 and AVPG-2 were 5.14 × 10^5^ Da and 1.48 × 10^7^ Da, respectively. Methylation and NMR analysis revealed that the backbone was → 4)-α-dD-Glc*p*-(1 → 3,4)-β-d-Glc*p*-(1 → 4)-α-d-Glc*p*-(1 → and → 4)-α-d-Glc*p*-(1 → 3,6)-β-d-Gal*p*-(1 → 4)-α-d-Glc*p*-(1 →, respectively. Biological activity study revealed that AVPG-2 could turn the polarization of macrophages to the M1 direction and increased cell viability, promoted the secretion of NO and cytokines and boosted the phagocytic capacity of macrophages. Therefore, AVPG-2 exhibited good immunomodulatory activity. AVPG-2 has the potential to act as a natural immunomodulatory ingredient or a functional food.

## Figures and Tables

**Table 1:** Results of the methylation analysis of AVPG-1 and AVPG-2.

Methylated Sugars	Linkage	AVPG-1 (Molar Ratios)	AVPG-2 (Molar Ratios)
2,3,6-Me_3_-Glcp	→4)-Glc*p*-(1→	0.65	0.36
2,6-Me_2_-Glc*p*	→3,4)-Glc*p*-(1→	0.16	ND
2,3,6-Me_3_-Gal*p*	→4)-Gal*p*-(1→	0.09	0.04
2,3,4-Me_3_-Xyl*p*	Xyl*p*-(1→	0.04	ND
2,3,5-Me_3_-Ara*f*	Ara*f*-(1→	0.06	0.09
1,2,3,4-Me_4_-Glc*p*	→6)-Glc*p*	ND	0.09
2,4,6-Me_3_-Gal*p*	→3)-Gal*p*-(1→	ND	0.03
2,4-Me_2_-Gal*p*	→3,6)-Gal*p*-(1→	ND	0.17
2,3-Me_2_-Ara*f*	→5)-Ara*f*-(1→	ND	0.06
2,3-Me_2_-Xyl*p*	→4)-Xyl*p*-(1→	ND	0.10
2,3,4-Me_3_-Rhal*p*	Rha*p*-(1→	ND	0.02

ND indicates not detected.

**Table 2:** ^1^H and ^13^C NMR chemical shifts (in ppm) for AVPG-1 and AVPG-2 recorded in D_2_O at 30 °C.

Peak	Glycosyl Residue	H-1/C-1	H-2/C-2	H-3/C-3	H-4/C-4	H-5/C-5	H-6/C-6
*Chemical shifts of residues of AVPG-1*							
A	-4)-α-d-Glcp-(1-	5.38/99.70	3.62/71.01	3.95/72.92	3.64/73.25	3.76/72.26	3.41, 4.03 /68.98
B	-3,4)-β-d-Glcp-(1-	4.45/101.61	3.35/72.80	3.80/80.18	3.74/73.55	3.56/73.40	3.70, 3.94 /60.44
C	β-d-Xylp-(1-	4.71/103.10	3.76/75.83	4.03/71.18	3.41/71.67	3.70, 4.12/67.49	
D	-4)-α-d-Galp-(1-	5.22/108.35	3.56/78.60	3.26/72.21	3.69/79.54	4.11/76.60	3.80, 4.00 /67.54
E	α-l-Araf-(1-	5.20/109.20	4.19/80.85	3.92/75.86	4.11/83.77	3.36, 4.20/62.46	
F	α-d-GlcpA-(1-	5.26/97.60	4.14/80.37	3.69/79.68	3.82/70.89	4.04/61.64	-/179.22
*Chemical shifts of residues of AVPG-2*							
A	-6)-β-d-Glcp	4.62/95.84	3.22/71.19	3.45/75.81	3.87/69.30	4.09/68.42	3.70, 3.94/61.23
B	-4)-α-d-Glcp-(1-	5.40/99.70	3.62/71.55	3.94/73.19	3.61/76.66	3.75/76.47	3.41, 3.98/68.70
C	-3)-α-d-Galp-(1-	5.39/101.63	3.62/71.43	3.70/79.94	3.40/69.51	3.93/73.11	4.12, 4.19/68.14
D	-4)-α-d-Galp-(1-	5.22/108.27	3.50/71.36	3.26/72.73	3.67/72.58	3.40/72.25	3.71, 3.82/60.94
E	-3,6)-β-d-Galp-(1-	4.41/103.09	3.50/71.63	3.70/80.03	3.53/73.81	3.36/69.57	3.71, 4.09/68.50
F	α-l-Araf-(1-	5.23/109.18	4.19/81.19	3.80/76.31	4.11/76.43	3.45, 3.80/60.36	
G	α-l-Araf-(1-	5.20/92.01	4.20/81.25	3.91/76.63	4.11/76.52	3.45, 3.80/60.36	
H	-5)-α-l-Araf-(1-	5.07/98.92	4.11/83.85	3.92/76.49	4.19/81.18	3.68, 3.91/76.67	
I	-4)-β-d-Xylp-(1-	4.49/102.72	3.34/73.17	3.55/73.97	3.94/76.49	3.39, 4.10/62.89	
J	α-l-Rhap-(1-	4.71/100.70	3.90/73.37	4.11/80.40	3.41/72.03	4.01/69.88	1.23/16.42
K	α-d-GlcpA-(1-	5.26/97.61	4.14/80.60	3.92/76.47	4.19/80.94	3.98/68.66	177.05
L	-4)-α-d-GalpA	5.06/107.43	3.74/79.84	3.63/71.49	4.10/83.89	3.92/76.46	175.38

## Data Availability

The data presented in this study are available in article and supplementary material.

## Supplementary Materials

The following are available online, Figure S1: FT-IR spectrum of the partially methylated AVPG-1, Figure S2: FT-IR spectrum of the partially methylated AVPG-2, Table S1: The cross signals obtained from HMBC spectra of AVPG-1 and AVPG-2.

## Sample Availability

Not available.
