# Supplementary material for: Purification, Structural Characterization and Immunomodulatory Effects of Polysaccharides from Amomum villosum Lour. on RAW 264.7 Macrophages
Source: Molecules. 2021 May 2;26(9):2672. doi: 10.3390/molecules26092672 (PMC8125432; doi:10.3390/molecules26092672)
Supplement: Supplementary file 1 [file molecules-26-02672-s001.zip › molecules-1157157-supplementary.pdf]

molecules

MDPI

Article

Purification and structural characterization of polysaccharides with potential immunomodulatory activities from *Amomum villosum* Lour.

Yang Zhou [1], Chunguo Qian [1], Depo Yang [1,2], Cailin Tang [1], Xinjun Xu [1,2], E-Hu Liu [3], Jingtang Zhong [4], Longping Zhu [1,2] and Zhimin Zhao [1,2,*]

[1] School of Pharmaceutical Sciences, Sun Yat-Sen University, Guangzhou 510006, China; zhouy596@mail2.sysu.edu.cn (Y.Z.); qianchg@mail2.sysu.edu.cn (C.Q.); lssydp@mail.sysu.edu.cn (D.Y.); tangclin@mail2.sysu.edu.cn (C.T.); xxj2702@sina.com (X.X.); zhlongp@mail.sysu.edu.cn (L.Z.)
[2] Guangdong Technology Research Center for Advanced Chinese Medicine, Guangzhou 510006, China
[3] State Key Laboratory of Natural Medicines, School of Traditional Chinese Pharmacy, China Pharmaceutical University, Nanjing 210009, China; liuehu2011@163.com
[4] Yayisan Chinese Herbs Plantation Ltd., Heyuan 517428, China; zhaozhimin1978@hotmail.com
* Correspondence: zhaozhm2@mail.sysu.edu.cn

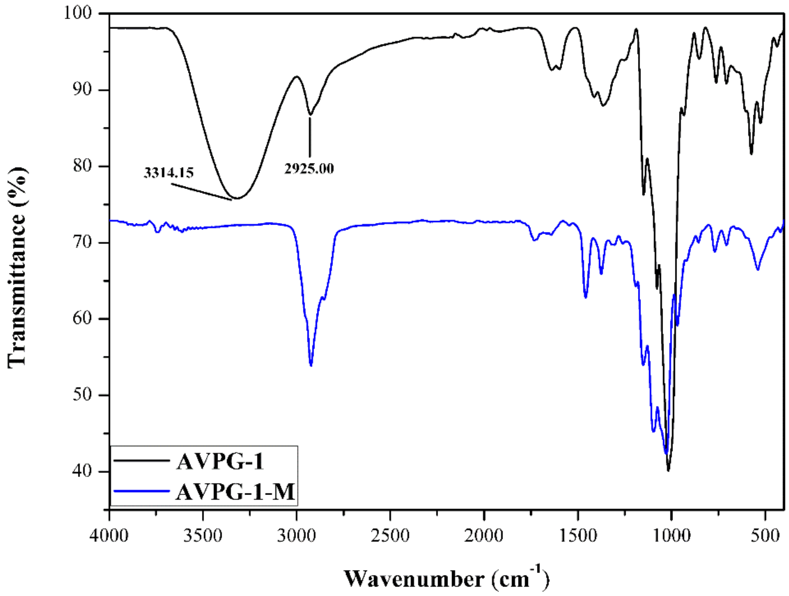

Figure S1. FT-IR spectrum of the partially methylated AVPG-1 (AVPG-1-M).

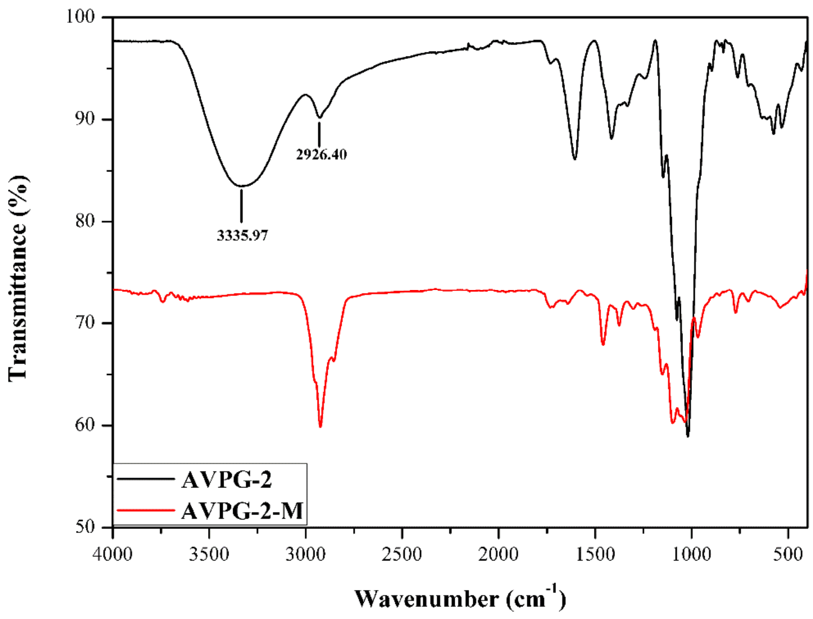

Figure S2. FT-IR spectrum of the partially methylated AVPG-2 (AVPG-2-M).

Table S1. The cross signals obtained from HMBC spectra of AVPG-1 and AVPG-2.

Residues	$^1H/^{13}C$	$\delta_{H/C}$	Residues	$^1H/^{13}C$	$\delta_{H/C}$	Sequences
The cross signals of AVPG-1						
A	H-1	5.38	A	C-4	76.14	$AC_4 \rightarrow AO_1$
A	H-4	3.64	A	C-1	99.70	$AC_1 \rightarrow AO_4$
A	H-1	5.38	B	C-4	75.53	$BC_4 \rightarrow AO_1$
A	H-4	3.64	B	C-1	101.61	$BC_1 \rightarrow AO_4$
D	H-4	3.69	A	C-4	76.14	$AC_4 \rightarrow DO_4$
C	H-1	4.71	B	C-3	80.18	$BC_3 \rightarrow CO_1$
A	H-4	3.64	D	C-4	79.54	$DC_4 \rightarrow AO_4$
A	H-1	5.38	E	C-1	109.20	$EC_1 \rightarrow AO_1$
D	H-1	5.22	B	C-3	80.18	$BC_3 \rightarrow DO_1$
E	H-1	5.20	A	C-1	99.70	$AC_1 \rightarrow EO_1$
F	H-1	5.26	A	C-1	99.70	$AC_1 \rightarrow FO_1$
The cross signals of AVPG-1						
B	H-1	5.40	B	C-4	76.66	$BC_4 \rightarrow BO_1$
B	H-4	3.61	B	C-1	99.70	$BC_1 \rightarrow BO_4$
E	H-3	3.70	B	C-1	99.70	$BC_1 \rightarrow EO_3$
B	H-4	3.61	E	C-1	103.09	$EC_1 \rightarrow BO_4$
E	H-6	4.09	I	C-4	76.49	$IC_4 \rightarrow EO_6$
I	H-4	3.94	E	C-6	68.50	$EC_6 \rightarrow IO_4$
I	H-1	4.49	A	C-6	69.37	$AC_6 \rightarrow IO_1$
A	H-6	3.39	I	C-1	102.72	$IC_1 \rightarrow AO_6$
D	H-4	3.67	E	C-6	68.50	$EC_6 \rightarrow DO_4$
D	H-1	5.22	C	C-3	79.94	$CC_3 \rightarrow DO_1$
C	H-3	3.70	D	C-1	108.27	$DC_1 \rightarrow CO_3$
C	H-1	5.39	H	C-5	73.02	$HC_5 \rightarrow CO_1$
H	H-1	5.07	F	C-1	109.18	$FC_1 \rightarrow HO_1$
J	H-1	4.71	E	C-6	68.50	$EC_6 \rightarrow JO_1$
E	H-6	3.71	H	C-1	98.92	$HC_1 \rightarrow EO_6$
H	H-5	3.68	L	C-4	83.89	$LC_4 \rightarrow HO_5$
E	H-6	3.71	B	C-1	99.70	$BC_1 \rightarrow EO_6$
K	H-1	5.26	B	C-4	76.66	$BC_4 \rightarrow KO_1$
E	H-6	4.09	B	C-4	76.66	$BC_4 \rightarrow EO_6$
G	H-1	5.20	B	C-1	99.70	$BC_1 \rightarrow GO_1$
